# It’s Time to Change Direction on Agility Research: a Call to Action

**DOI:** 10.1186/s40798-021-00304-y

**Published:** 2021-02-12

**Authors:** Warren Young, Russell Rayner, Scott Talpey

**Affiliations:** 1 School of Science, Psychology and Sport, Federation University Australia, Ballarat, Victoria Australia; 2 School of Health and Sport Science, Eastern Institute of Technology, Napier, Hawke’s Bay New Zealand

**Keywords:** Agility, Change-of-direction, Research approaches, Invasion sports

## Abstract

Agility is an important skill for both attackers and defenders in invasion sports such as codes of football. On the sporting field, agility requires reacting to a stimulus, often presented by an opponent’s movement, before a change of direction or speed. There is a plethora of research that examines the movement component of agility in isolation, which is described as change-of-direction (COD) ability, and this is thought to underpin agility performance. This opinion article proposes that COD ability should not be researched as the only or primary outcome measure when the objective is to inform agility performance in invasion sports. It is argued that pre-planned COD movements and tests lack ecological validity because they lack perception-action coupling and involve movement out of context from the game. The movement techniques and strength qualities required for the performance of COD tests can be quite different to those required for agility. It is suggested that COD tests can be applied to sports that involve pre-planned COD movements, but researchers should endeavour to use agility tests when studying invasion sports. Some new methods for assessing one-on-one agility contests are reported as potentially valuable for future research, and examples of research questions are provided.

## Key Points

- Change-of-direction (COD) ability is widely researched and often the findings are claimed to apply to invasion sports such as codes of football
- COD ability is an example of an isolated component of agility in which the movement and perception are decoupled, and therefore, COD tests can lack ecological validity
- The movement techniques and strength qualities that are important in many COD movements do not necessarily apply to agility in invasion sports

## Introduction

Agility is a skill considered to be important for many sports and has been defined as “a rapid whole-body movement with change of velocity or direction in response to a stimulus” [1]. Although there are alternative definitions in the sport science literature, it is generally accepted that agility has both movement and reactive elements, whereas predetermined change-of-direction (COD) tasks without a requirement to react to a stimulus are described as COD ability [2].

In invasion sports such as all codes of football, agility is important for an attacker to evade defenders to maintain possession. It requires the use of diverse footwork strategies performed at various speeds and angles and may involve deceptive actions. Conversely for defenders, agility is important to allow players to move quickly and accurately to block attackers, with the ultimate objective of taking possession of the ball. Therefore, in invasion sports, agility is a complex and varied open skill.

Pre-planned COD tasks are present in some sports. For example, cricket batters can score more than a single run by performing a 180° turn at each end of the pitch. To perform this effectively, the player must visually target the crease, adjust the steps, and decelerate before stopping and reaccelerating in the opposite direction. It is a movement whereby the choice of technique that will be performed and the location and timing of the movement can be pre-planned. This stereotypical COD scenario has been replicated and assessed with a test such as the “505” [3]. However, most other COD tests do not target the footwork demands of particular sports and appear to be rather generic in nature. An example is the Illinois agility test, which requires the athlete to execute nine changes of direction by weaving around cones in a predetermined path as fast as possible. Tests requiring multiple changes of direction around obstacles may also be described as tests of “manoeuvrability” [4].

## Pre-planned COD and Agility Are Different

In invasion sports, pre-planned COD scenarios either do not occur in competition, or are extremely rare. Although football codes do involve set pieces or pre-planned routes, these reflect a tactical strategy rather than an actual agility scenario. Attacking players may plan the direction they wish to run, as well as an angle of cut, but when the athlete is faced with a defender in close proximity, the exact footwork and angle of cut can be variable, depending on the situation. The attacker may attempt to lead a defender in one direction with a deceptive action, but may need to instantly change the movement strategy if the deceptive action is not successful. Likewise, a defender has to constantly scan the attacker’s motion to react as quickly and accurately as possible. Evidence exists that attacking and defending agility are relatively independent skills [5, 6]. This highlights the complexity, variability, and unpredictability of agility displayed on the sporting field. It also explains why a pre-planned COD task is unlikely to reflect the agility demands of competition in invasion sports.

To understand agility, it is appealing for sport scientists or researchers to deconstruct this skill into smaller, more manageable components. Such a reductionist approach is thought to simplify the assessment and the training of this quality. One way to achieve this is to identify the many factors that explain agility performance. For example, a deterministic model was published in 2002 [7], which indicates that agility is comprised of two subcomponents: COD speed and a perceptual and decision-making component, which are in turn determined by many other factors. This model has been referenced by researchers [8, 9] to justify the notion that COD ability underpins agility performance, and therefore, studying COD in isolation can be useful for understanding agility performance in a range of invasion sports [10–13].

Although not explicitly stated by researchers, another likely reason for the plethora of research in COD ability is the relative ease in assessing it and quantifying performance. The appeal of such testing relates to minimal equipment needed, time-efficiency for assessing large numbers of participants, readily available normative data, and good reliability due to the standardisation of the test protocols. Further, since biomechanical research indicates that pre-planned sidestepping movements place less load on the knee compared to sidestepping requiring reaction to a stimulus [14], another perceived advantage of COD testing is a reduced risk of injury such as an anterior cruciate ligament rupture.

By contrast, assessment of agility is more complex because of the requirement to react to a stimulus. Some researchers have acknowledged the need to include a stimulus to assess agility, and have used a flashing light device to direct the athlete to a new location [15–19]. While this seems like a relatively simple and easily controlled solution, this type of “generic” stimulus is non-existent in the sporting environment and therefore continues to lack ecological validity. Research that has compared a generic stimulus to a more sport-specific stimulus of an opponent’s movement has shown that the former fails to discriminate higher and lower standard athlete groups [20–22]. Further, a light-based generic stimulus evokes a different side-stepping technique compared to a sport-specific stimulus [23, 24].

## Moving Towards Research with Agility Outcome Measures

A key feature of this article is to question the notion of studying COD ability in isolation, and we appeal to researchers to focus on agility performance, with the objective of improving the ecological validity of the research. We suggest that COD research findings cannot automatically be extrapolated to agility in invasion sports. It is hoped that a change of direction in the focus of research may help with an improved understanding of agility performance as it is played out on the sporting field.

We propose a number of reasons for moving away from COD research. First, while some athletic skills such as linear sprinting and jumping may lend themselves to a reductionist analytical approach, agility is arguably far more complex and variable and therefore may be better understood with a more holistic approach. Since agility involves movement in response to a stimulus, it requires a “perception-action coupling”, that is, the movement and perception (stimulus) influence each other, and to maintain the integrity of the skill, should not be separated or isolated. A decoupling of the demands on the perceptual and motor subsystems during practice would not be commensurate with the establishment of cortical networks for perceiving information for movement [25, 26]. Therefore, according to Davids and co-workers [27], rather than decoupling or decomposing a complex skill into parts, it is preferable to simplify the skill to facilitate learning. In relation to agility, this may be achieved by reducing the speed of movement or the number of opponents. When a pre-planned COD movement is trained in isolation from agility, it may be considered as part practice. Since this movement is out of context from the sporting field, it has different sensorimotor information to an agility task requiring reaction to a stimulus [28]. According to Bosch [28], this part practice has lower transferability to the whole agility skill. Therefore, analysing COD in isolation with the view of improving its performance may be a misguided strategy for enhancing agility performance.

Second, according to the concept of perception-action coupling, technique changes when reacting to a stimulus. A systematic review and meta-analysis of sidestepping [14] shows convincingly that several biomechanical variables describing sidestepping technique are significantly different when reacting to a stimulus, when compared to a pre-planned COD task. Such findings in a rugby context led Wheeler and Sayers [29] to conclude that the absence of decision-making elements in agility testing and training programmes may result in the incorrect movement patterns being trained. The dissimilarity of technique is likely to be one reason why COD and agility skills have been shown to possess relatively low statistical commonality [22, 30–32], and have been described as relatively independent qualities [33].

Thirdly, factors considered important for the enhancement of COD ability may be different for agility. By determining the role of strength qualities for performance in COD tasks, researchers seek to inform strength and conditioning practice. However, it should not be assumed that the physical qualities that are important for COD performance are equally as relevant to agility. For example, Spiteri et al. [34] reported that various tests of lower body strength qualities correlated significantly with COD performance, but not with an agility test in female basketball players. Similarly, Young et al. [35] found a large correlation between reactive strength and a COD task (*r* = − 0.645, *P* < 0.01), whereas only a small association was found for an agility task with a similar movement pattern (*r* = − 0.101, *P* > 0.05). The authors suggested that in the defensive agility manoeuvre, the participants appeared to use small steps, which may not have allowed the athletes to express their reactive strength as well as in the pre-planned COD task. It was also speculated that the presence of the decision-making element in the agility test may have diminished the importance of the strength qualities, compared to the COD task. It would be valuable to determine if the development of strength qualities that can enhance COD performance [2] can transfer to agility performance, but to our knowledge, training studies answering this question are non-existent.

Based on the above discussion, we propose that the findings of COD research should not be assumed to apply directly to on-field agility, such as in invasion sports. Research on COD ability is best applied to sports requiring pre-planned COD tasks, such as running between wickets in cricket, or between bases in baseball and softball. However, if researchers wish to make positive contributions to agility performance as required in invasion sports, they should endeavour to assess sport-specific agility as an outcome measure.

## Assessing Agility

Research informing practice relating to agility performance may be enhanced when more ecologically valid testing methods are adopted. One option that has appeared in the recent sport science literature is assessing agility in a one-versus-one (1 v 1) scenario [5, 6, 36]. These tests have attempted to create an agility scenario similar to a contest between an attacker and defender in rugby or Australian football. An example is where the attacker is instructed to attempt to evade a defender to cross an end-line without being tagged, while the defender is instructed to attempt to tag the attacker’s torso with two hands. Scoring is graded according to the proximity of each player to each other in the contest, which may be confirmed by video footage [5]. These tests have been shown to have acceptable reliability [5, 6, 36], and appear to have good face validity and ecological validity. Some potential advantages of these tests include retention of perception-action coupling, similar footwork and movement patterns to on-field competition, variability of COD techniques, movement speeds and perceptual information, and the capacity to assess both attacking (including deceptive actions) and defending agility. A limitation of this approach is the restricted perceptual information associated with reacting to just one opponent. Although this scenario may be common in many sports, there are also many occasions where an attacker or defender needs to react to multiple opponents, or at least consider the actions of many players on the field or court. Researchers have attempted to include multiple opponents [23, 24], although the application was related to the injury risk of sidestepping rather than performance. Another potential method that should be explored to assess agility performance is the use of virtual reality technology.

A further step towards assessing agility performance in a naturalistic setting is with small-sided games (SSG). While these activities are thought to be effective for training sport-specific agility [37, 38], SSG have not yet been used to assess agility performance. The inherent variability of agility in games poses a significant challenge to the development of a reliable test. In the absence of suitable quantitative outcome measures, a potential alternative might be to describe agility performance qualitatively [4] via a checklist of technical considerations and outcomes of contests in games.

## Conclusions

In summary, we argue that researchers should not focus on COD ability as the key outcome measure when interested in informing practice relating to agility in invasion sports. It should not be assumed that the training methods and test protocols applicable to COD ability will automatically be suited to agility performance. Instead, we encourage researchers to study agility in a more naturalistic setting that can be expected to transfer to on-field performance. Due to the inherent complexity and variability associated with agility, this may present methodological challenges. The use of a 1 v 1 contest for agility assessment in invasion sports appears to be a promising step in this direction, but more research is needed to evaluate this approach. Researchers should also explore other assessment methodologies such as virtual reality, SSG, and the use of qualitative descriptions of performance. These or similar approaches could be used to address some potential research questions:

- Can existing agility tests be modified for other sports, while maintaining acceptable reliability?
- Do various methods of resistance training enhance agility performance?
- What agility techniques are most successful for agility performance?
- What is the best way to schedule training sessions to enhance agility skill?
- How does the manipulation of constraints in 1v1 or small-sided games influence agility development?
- What deceptive strategies are most effective for evasion by an attacker?
- What perceptual information is used by highly agile performers in attacking and defending roles?
- How does agility skill relate to on-field team performance indicators such as successful tackles and team possession?

As a final note, we are not suggesting that there is no place for isolating agility technique in training. Indeed, it is likely that sport-specific COD technique training provides a foundation for more complex agility skills in a periodised programme. However, it is important that researchers seek to assess agility skill that can be demonstrated to be relevant to on-field performance.

## Data Availability

Not applicable

## Abbreviations

COD: Change-of-direction
1 v 1: One-versus-one
